# French Gratitude to Medical Men

**Published:** 1844-01

**Authors:** 


*French Gratitude to Medical Men.*—We observe by one of the Paris journals, that the names of *Percy*, *Desgenettes*, and *Larrey*, have been recently engraved on the famous Arc de Triomphe, at the Barriére de l'Etoile.

In some respects, certainly, medical men occupy a higher position in society in France than in this country. For example, who ever heard of a doctor becoming a *Baron*, or peer of the realm with us? and yet every one knows that *Portal*, *Dupuytren*, and *Cuvier*, occupied that dignified rank in the parliament of France; and where is the nobleman of Germany that might not be proud to be associated with a Baron *Humboldt?* True, the circumstance of a French peerage being merely personal, and not hereditary, makes a very material difference in the case; but then, why should there not be a certain number at least—we are no friends to an elective aristocracy, as a general question—of the most eminent men in the various departments of science being admitted to the highest honors of the state, without necessarily entailing upon their families the expensive title which they bore themselves?

By a recent Royal Ordonnance, M. *Louis* has been made an officer of the Legion of Honor, and M. *Leuret* a chevalier of the same Order.

Again, MM. *Andral* and *Rayer*—certainly two of the most distinguished names in French medicine of the present age—have been elected members of the Institute. "The selection of such men," says one of the Paris jouınals, "shows very emphatically that this illustrious body looks as much to the scientific character as to the practical eminence of its candidates; in a word, that it wants not so much the mere skilful physician as the enlightened philosopher in its ranks."—*Medico-Chirurgical Review for July*, 1843.

